# Role of contrast-enhanced ultrasound in assessing indeterminate renal lesions and Bosniak ≥2F complex renal cysts found incidentally on CT or MRI

**DOI:** 10.1259/bjr.20210707

**Published:** 2021-08-31

**Authors:** Giuseppe Como, Claudio Valotto, Francesco Tulipano Di Franco, Gianluca Giannarini, Lorenzo Cereser, Rossano Girometti, Chiara Zuiani

**Affiliations:** 1 Institute of Radiology, Department of Medicine, University of udine, University Hospital S. Maria della Misericordia, Udine, Italy; 2 Urology Unit, University Hospital S. Maria della Misericordia, Udine, Italy

## Abstract

**Objective:**

To investigate the impact of contrast-enhanced ultrasound (CEUS) in reclassifying incidental renal findings categorized as indeterminate lesions (IL) or Bosniak ≥ 2F complex renal cysts (CRC) on CT or MRI.

**Methods:**

We retrospectively included 44 subjects who underwent CEUS between 2016 and 2019 to assess 48 IL (*n* = 12) and CRC (*n* = 36) incidentally found on CT or MRI. CEUS was performed by one radiologist with 10 year of experience with a sulfur hexafluoride-filled microbubble contrast agent. The same radiologist, blinded to clinical information and previous CT/MRIs, retrospectively reviewed CEUS images/videos, categorizing renal findings with Bosniak-derived imaging categories ranging from 0 (indeterminate) to 5 (solid lesion). CEUS-related reclassification rate was calculated (proportion of IL reclassified with an imaging category >0, or CRC reclassified below or above imaging category >2F). Using histological examination or *a* ≥ 24 months follow-up as the standard of reference, we also estimated per-lesion sensitivity/specificity for malignancy.

**Results:**

CEUS reclassified 24/48 findings (50.0%; 95% C.I. 35.2–64.7), including 12/12 IL (100%; 95% CI 73.5–100) and 12/36 CRC (33.3%; 95% C.I. 18.5–50.9), mostly above category >2F (66.7%). CEUS and CT/MRI showed 96.0% (95%CI 79.7–99.9) *vs* 44.0% (95%CI 24.4–65.1) sensitivity, and 82.6% (95%CI 61.2–95.1) *vs* 60.9% (95%CI 38.5–80.3%) specificity.

**Conclusion:**

CEUS provided substantial and accurate reclassification of CT/MRI incidental findings.

**Advances in knowledge:**

Previous studies included Bosniak 2 incidental findings, thus possibly underestimating CEUS-induced reclassification rates. Using a more meaningful cut-off (Bosniak ≥2F), problem-solving CEUS was effective as well, with higher reclassification rates for CRC than in literature.

## Introduction

Over the last years, improved imaging technology led to increased incidental detection of renal findings in patients undergoing CT and/or MRI examinations for reasons unrelated to the genitourinary system.^1^ CT and MRI are accurate in characterizing and driving the management of most incidental findings. However, sometimes CT and MRI findings can be ambiguous. Some incidental findings present as indeterminate lesions (IL) with unclear solid *vs* cystic nature,^2,3^ thus prompting the problem of how to further differentiate and manage them.^4^ Moreover, up to 8% of incidental findings are complex renal cysts (CRC) with difficult Bosniak categorization on CT or MRI.^5^ In particular, the main difficulty is differentiating Bosniak 2F *vs* Bosniak 3 category. While about 50% of Bosniak 3 category findings are benign,^6^ reliable differentiation between 2F and 3 category should be provided in order to tailor patient management.

Contrast-enhanced ultrasound (CEUS) is gaining ever-increasing acceptance as a tool to assess renal lesions, given the rapidity of execution, reduced costs, safe contrast medium profile, and promising clinical results.^7,8^ By using a microbubble contrast-agent, CEUS is able to characterize IL by differentiating even minimally vascularized solid lesions from avascular cysts, thus helping in addressing them to proper management.^3,7^ CEUS also showed better accuracy than CT in assessing Bosniak categorization-related features such as internal septa, parietal thickening, mural nodules, and contrast-enhancement.^9,10^ A recent metanalysis by Furrer et al^11^ showed 85 *vs* 79% pooled accuracy in characterizing complex renal cysts for CEUS *vs* CT/MRI, respectively. Several studies also found CEUS to be effective in assessing malignant incidental renal findings, with approximately 95% accuracy for masses presenting as indeterminate on CT,^3^ 96% sensitivity/100% specificity for masses of unclear solid nature on MRI,^12^ and 100% sensitivity/96.6% specificity for indeterminate or cystic renal lesions found on ultrasound, CT or MRI.^13^

However, to our knowledge current evidence on CEUS has been acquired including incidental findings initially classified as Bosniak 2 by CT or MRI (up to 58.7% of CRC in previous studies^14^). This might have underestimated the impact of CEUS, as Bosniak 2 observations are easy-to-characterize findings with low likelihood of reclassification. Consequently, little is known on the role for CEUS in a population of incidental findings including Bosniak category ≥2F as a more meaningful CRC cut-off for prompting a problem-solving examination.

On this basis, the purpose of this study was to determine the impact of CEUS in reclassifying incidental renal findings initially assessed as IL or CRC with Bosniak category ≥2F.

## Methods and materials

### Study population and revision of previous imaging

This study was approved by the referring Institutional review board. Because of the retrospective design, the acquisition of written informed consent was waived.

We reviewed patients records to identify all subjects who, in accordance with our institutional policy, underwent CEUS to further asses renal lesions found incidentally in previous CT or MRI examinations performed for indications unrelated to the genitourinary system.

The period of search was February 2016 to October 2019. Of 77 eligible patients, we included those with a CT or MRI examination performed within 3 months before CEUS, showing one or more renal findings assessed as IL or CRC with Bosniak classification at least 2F.^6,15^ In accordance with the American College of Radiology definition,^16^ a renal finding was considered as indeterminate when difficult to diagnose as benign or malignant at the time of primary imaging, *e.g*. because of indistinguishable solid *vs* cystic pattern. Other inclusion criteria were the availability of the standard of reference as defined below, and having undergone an image revision confirming the indeterminate nature of renal findings. Image revision was performed by a study coordinator with 10 years of experience in abdominal imaging, blinded to CEUS results, who reviewed CT and MRI examinations to assess technical adequateness and confirm renal findings as IL or CRC. CT examinations were assessed as adequate if performed with contrast administration on *a* ≥ 16 row scanner with reconstructed slice thickness ≤2.5 mm. MRI were assessed as adequate if performed on *a* ≥ 1.5 T magnet, being inclusive of at least transverse in-phase/out-of-phase *T*_1_ weighted imaging, fat-suppressed *T*_2_ weighted imaging, and diffusion-weighted imaging (DWI). When both CT and MRI were available, the coordinator included in the analysis MRI in the case of IL, and the examination with the highest Bosniak score in the case of CRC. CT criteria to confirm a lesion as IL were attenuation values in the range 20–70 Hounsfield units (HU) on unenhanced scan, combined with homogeneous appearance and/or equivocal contrast-enhancement after intravenous contrast administration (increase in attenuation ≤20 HU). No lesion was assessed as IL based on MRI appearance. All patients were confirmed with IL or CRC requiring problem-solving CEUS. We excluded 33 patients with no standard of reference available (see below).

Final population included 44 subjects (33 males, 11 females) with a mean age of 68.1 years (range 36–90 years), and a total of 48 renal index lesions. Index lesion was defined as the imaging finding prompting CEUS. Patients have previously performed a total of 47 examinations, *i.e*. CT alone in 32/44 cases, MRI alone in 9/44 cases, and both CT and MRI in the remaining 3/44 cases. A total of 17 examinations have been performed outside the Institute of Radiology, University of Udine, University Hospital S. Maria della Misericordia, Italy.

### CEUS technique

CEUS examinations were performed during clinical routine by a single radiologist with 20 years of experience in abdominal ultrasound (10 years specifically in CEUS), unblinded to previous CT and/or MRI findings, using a Logiq E9 system (General Electric Healthcare, Milwaukee, WI, USA). This radiologist did not correspond to the study coordinator.

After preliminary grayscale and color Doppler investigation, CEUS was set with low acoustic power to achieve minimum microbubble destruction (mechanical index of 0.12). Each lesion confirmed by preliminary ultrasound investigation (100%) was evaluated separately after intravenous administration of a 2.4 ml dose of a sulfur hexafluoride-filled microbubble contrast agent (SonoVue, Bracco, MIlan, Italy), followed by a 10 ml saline flush. Per-index lesion digital cine-clips were acquired from the start of contrast injection to at least 2 min thereafter, together with static images, and sent to the picture archiving and digital system (PACS) (Suitestensa, Ebit AET, Genova, Italy) for subsequent review.

### Image analysis and standard of reference

Starting from late 2020, the same radiologist who performed CEUS examination reviewed images and videos stored in the PACS, blinded to the original report, as well as to CT and MRI images and clinical history. We did not use original CEUS reports for analysis, as this would have implied unblinding to those data. Reader was aware only of the side and site of the incidental finding to be reviewed.

During each reading session, IL and CRC to review were shown to him by the study coordinator. For the purpose of analysis, the radiologist categorized renal findings according to the above-mentioned criteria reported in Table 1, i.e. using a modified version of the Bosniak classification including imaging category 0 (IL) and imaging category 5 (findings presenting as solid). Image analysis did not include time–intensity curves (TIC) plotting contrast enhancement over time, as they are not part Bosniak categorization. Moreover, the focus on differential diagnoses for which TIC might represent a contribution was beyond the purpose of this study.

**Table 1.** Sonographic criteria used to categorize renal lesions on CEUS (adapted from Schoots et al^17^)

Imaging category	Definition	Findings on B-mode ultrasound	Contrast-enhanced ultrasound criteria
**0**	Indeterminate solid versus cystic lesion	Unspecific, *i.e*. without the appearance of a tumor or cyst	No contrast enhancement
**1**	Simple cyst = Bosniak 1	Thin walls, and no septa, and sharp margins, and no calcifications and/or solid components	No contrast enhancement
**2**	Minimally complex cyst = Bosniak 2	Septa thinner than 1 mm, with or without subtle calcifications	No contrast enhancement, or slight contrast enhancement of the septa
**2F**	Slightly complex cyst = Bosniak 2F	Multiple septa, and/or minimally thickened walls, and/or thin or thick calcifications	Moderate contrast enhancement of the walls and/or septa
**3**	Complex cyst = Bosniak 3	Homogenous or irregular thickening of the walls and/or septa, and/or irregular calcifications	Contrast enhancing septa and/or thickened wall
**4**	Mixed solid-cystic lesion = Bosniak 4	Homogenous or irregular thickening of the walls and/or septa, and/or irregular calcifications, and/or solid components	Contrast enhancing septa, and/or irregular contrast enhancing soft tissue components
**5**	Solid lesion	Homogeneous or heterogeneous echoic soft tissue, with or without anechoic components	Diffuse, homogeneous enhancement

CEUS, contrast-enhanced ultrasound.

The standard of reference was pathological examination in lesions referred to surgery, as well as histological examination after biopsy. For index lesions assessed as benign by CEUS, the surrogate standard of reference was stability over an imaging follow-up of at least 24 months (median 27 months, range 24–39 months), defined as absent changes in appearance and size.

### Data analysis

Quantitative data were presented with descriptive statistics, using mean (± standard deviation) or median (with interquartile range; IQR) values, after checking for data normality with the Shapiro-Wilk test. Notable proportions were coupled with 95% confidence intervals (95% CIs).

We first assessed the reclassification rate, calculated as the proportion of changes in imaging categorization provided by CEUS as compared to CT and/or MRI over the total number of lesions, IL alone, and CRC alone. The threshold for reclassification was set imaging category >2F *vs* imaging category ≤2F in the case of CRC. For IL, we assessed as reclassified any lesion in which CEUS attributed an imaging 1–5 category according to what reported in Table 1. IL categorized as 0 on CEUS were considered as persistently indeterminate/non-reclassified. The agreement between CT/MRI and CEUS in assessing imaging categories was investigated with Cohen’s κ (k) statistic. Reference k values were as follows: 0.01–0.20 slight agreement; 0.21–0.40 fair agreement; 0.41–0.60 moderate agreement; 0.61–0.80 substantial agreement; and 0.81–0.99 almost perfect agreement.^18^

After matching imaging results with the standard of reference and surrogate standard of reference, we calculated the per-imaging category cancer detection rate (CDR) of CT/MRI or CEUS. CDR was defined as the percent ratio between the number of malignant cases and the total number of assignments in that category. Analysis was performed on a per-index lesion basis.

Finally, we calculated sensitivity and specificity for malignancy of CEUS and CT/MRI, using image category >2F as the cut-off.

Analysis was performed with a commercially available software (MedCalc Software bvba v. 18.11.6, Ostend, Belgium), using an α value of 0.05.

## Results

### Lesions characteristics and categorization by CT or MRI

Of initial examinations, 26/35 CT studies were performed with a quadriphasic protocol including unenhanced scan, late arterial phase, portal venous phase, and delayed phase 3–5 min after contrast injection. All the remaining CT examinations lacked one or more of the above phases, except for the portal venous phase. MRI studies were performed without contrast administration in the three cases associated with CT. All the remaining 9/12 standalone MRI examinations included a quadriphasic post-contrast scan.

Of 48 lesions referred to CEUS, 12 were IL (25.0%) and 36 were CRC (75.0%). Median (IQR) lesion size on CT or MRI was 26.5 mm (10–45 mm) for all lesions, 27.8 mm (16–47 mm) for IL alone, and 26 mm (10–55 mm) for CRC alone. Of 44 patients, 3 showed two unilateral lesions, 1 presented two bilateral lesions, while 40 presented one single unilateral lesion.

By definition (see above), all IL were categorized 0 with CT and MRI, while CRC were categorized 2F, 3 and 4 in 16/36 (44.4%), 12/36 (33.3%) and 8/36 (22.2%) cases, respectively.

### Reclassification by CEUS

According to CEUS, no findings were categorized as indeterminate (score 0). IL were assigned category 2 in 2/12 cases (16.7%), category 3 in 1/12 cases (8.3%), category 4 in 2/12 cases (16.7%), and category 5 in the remaining 7/12 cases (58.3%) respectively. CRC were attributed imaging category 1 in 4/36 cases (11.1%), category 2 in 5/36 cases (13.9%), category 2F in 9/36 cases (25.0%), category 3 in 9/36 cases (25.0%), category 4 in 5/36 cases (13.9%), and category 5 in 4/36 cases (11.1%), respectively. Overall, CEUS categorized renal findings > 2F in 28/48 cases.

**Figure 1.**
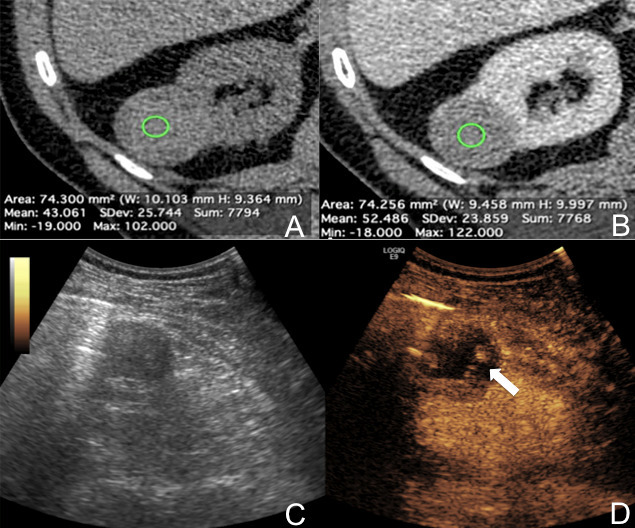
CEUS-induced reclassification to Bosniak 3 cyst of a right-sided IL found on CT. The round renal mass with partially ill-defined margins showed soft-tissue attenuation on basal unenhanced scan (values in the circular ROI in A). However, no significant contrast-enhancement was found, as shown by an increase in attenuation values < 20 HUs (values in the ROI in B). B-mode ultrasound (**C**) found a largely anechoic lesion, with multiple, small solid nodules better appreciated as enhancing areas after microbubble contrast administration (arrow in D). The lesion was proven to be clear cell renal carcinoma after surgical resection. CEUS, contrast-enhnaced ultrasound; HU, Hounsfield unit; IL, indeterminate lesion; ROI, region of interest.

**Figure 2.**
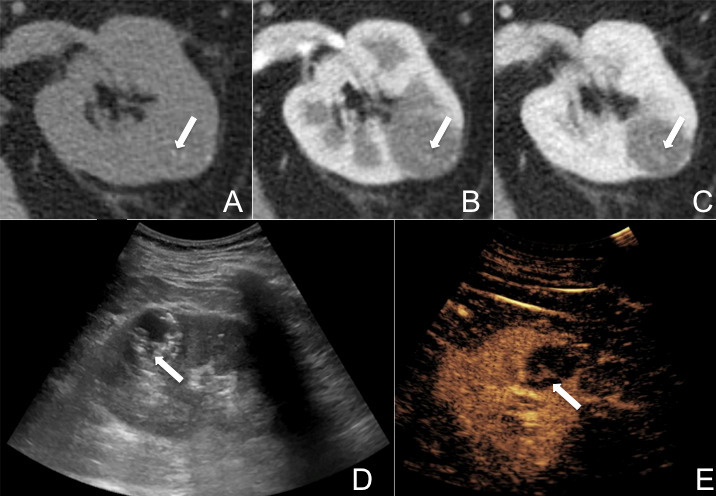
Reclassification of a CRC from Bosniak 2F (initial CT categorization) to Bosniak 3 category (final CEUS categorization). CT found a well-defined round mass in the left kidney, showing fluid attenuation and thin incompletely calcified septa on both unenhanced scan (arrow in A) and post-contrast arterial and venous phases (arrows in B and C). On preliminary B-mode ultrasound, a complex cysts with hyperechoic components was found (arrow in D), some of which showed measurable enhancement during CEUS evaluation (arrow in E). The lesion was referred to surgical treatment with pathological diagnosis of multilocular cystic renal neoplasm of low malignant potential. CEUS, contrast-enhnaced ultrasound; CRC, complex renal cyst.

Details on reclassifications are shown in Table 2. CEUS reclassified 24/48 CT/MRI findings (50.0%; 95% CI 35.2–64.7), including 12/12 IL (100%; 95% CI 73.5–100) and 12/36 CRC (33.3%; 95% CI 18.5–50.9) (Figure 1 and Figure 2). Most of them occurred towards imaging category >2F (16/24; 66.7%).

**Table 2.** Overview of cases reclassified by CEUS *vs* CT and MRI

	Reclassification towards category >2**F** (*n* = 16)			Reclassification towards category ≤2**F** (*n* = 8)		
	Initial CT/MRI category	Post-CEUS category	Number of reclassified cases	Initial CT/MRI category	Post-CEUS category	Number of reclassified cases
**CRC**	2F	3	5/36	-	-	-
	2F	4	1/36	-	-	-
	-	-	-	3	2	2
	-	-	-	3	2F	3
	-	-	-	4	2F	1
**IL**	0	3	1/12	-	-	-
	0	4	2/12			
	0	5	7/12	-	-	-
	-	-	-	0	2	2

CEUS, contrast-enhanced ultrasound; CRC, complex renal cyst; IL, indeterminate lesion.

The agreement between CT/MRI and CEUS in assigning imaging category >2F *vs* imaging category ≤2F was slight (*k* = 0.12) for all lesions, and fair (*k* = 0.33) when CRC only were considered.

### CDR

The CDR for each imaging category attributed by CT or MRI *vs* CEUS is shown in Table 3.

**Table 3.** Overview of cancer prevalence on a per-imaging category basis, as assessed by CT//MRI or CEUS

		Per-imaging category CDR						
		0	1	2	2F	3	4	5
**All lesions**	CT/MRI	10/12 (83.3%)	-	-	5/18 (27.8%)	4/10 (40.0%)	6/8 (75.0%)	-
	CEUS	-	0/4 (0%)	0/7 (0%)	1/9 (11.1%)	8/10 (80.0%)	6/7 (85.7%)	10/11 (90.9%)
**IL**	CT/MRI	10/12 (83.3%)	-	-	-	-	-	-
	CEUS	-	-	0/2 (0%)	-	1/1 (100%)	2/2 (100%)	7/7 (100%)
**CRC**	CT/MRI	-	-	-	5/18 (27.8%)	4/10 (40.0%)	6/8 (75.0%)	-
	CEUS	-	0/4 (0%)	0/5 (0%)	1/9 (11.1%)	7/9 (77.8%)	4/5 (80.0%)	3/4 (75.0%)

CDR, cancer detection rate;CEUS, contrast-enhanced ultrasound;CRC, complex renal cyst; IL, indeterminate lesion.

Among 28 findings categorized >2F by CEUS, 26/28 were surgically removed. Of them, 24/26 were found to be malignant (5 papillary renal cell carcinoma [RCC] Type 2, 6 papillary RCC Type 1, 1 papilary RCC solid/tubular variant, 10 clear cell carcinomas, 1 multilocular cystic neoplasm of low malignant potential and 1 high grade papillary urothelial carcinoma), while 2/26 were benign (1 renal oncocytoma and 1 focal segmental glomerulosclerosis, previously categorized 3 and 4 by CT, respectively). The remaining 2/28 lesions were referred to surveillance with CEUS, given severe comorbidity contraindicating intervention. None of them showed changes over time, so that they were categorized as false-positives.

One of the lesions categorized ≤2F by CEUS showed an increase in size (over 50%) at a CT scan performed at 25 months from the baseline CEUS, as confirmed by subsequent CEUS evaluation (where it was recategorized as 4). This false-negative case was finally proven to be papillary renal carcinoma by surgery. No other lesions categorized ≤2F by CEUS were found to be malignant at follow-up.

Sensitivity for malignancy of CEUS and CT/MRI was 96.0% (95%CI 79.7–99.9) *vs* 44.0% (95%CI 24.4–65.1), while specificity was 82.6% (95%CI 61.2–95.1) versus 60.9% (95%CI 38.5–80.3%), respectively.

## Discussion

In our study, CEUS reclassified 50% of incidental renal lesions found on CT and/or MRI, being able to define whether IL were solid or cystic in 100% of cases, and changing the Bosniak classification in about one-third (33.3%) of CRC. Of note, the large majority of lesions classified category >2F by CEUS were found malignant at final pathology (24/28), with 96% of malignant cases categorized 3–5. These results suggest that, when available, CEUS might be used for systematic target analysis of incidental IL and CRC found at CT and/or MRI, as the probability of reclassification and ruling-in malignancy is high. While this might increase costs and complexity of the diagnostic pathway, it is reasonably more effective, less costly, and at a lower risk of delaying relevant diagnoses than a CT and/or MRI follow-up of IL or CRC incorrectly categorized ≤2F.

Findings initially assessed as 2F by CT and/or MRI were the main object of reclassification among CRC, which occurred in 72.2% of assignments, either on the side of downgrading or upgrading (52.8 and 46.2% of the reclassified cases, respectively). This is in accordance with previous results by Schwarze et al,^19^ and matches with the expectedly high sensitivity of CEUS we observed.^13,20–22^ The reclassification rate we found in the CRC group was higher compared to what previously shown (10–20%).^9,14,23^ A possible explanation is that, differently from our work, previous studies included Bosniak category 2 cysts (prevalence up to 58.7%), thus potentially underestimating the reclassification impact of CEUS. Indeed, category 2 findings are easy to categorize, and have a reasonably low probability of being reclassified. This might also explain why CT and/or MRI showed lower sensitivity, specificity and accuracy compared to previous works,^22,24^ except a study by Defortescu et al^25^ which included Bosniak 2F and 3 observations only (sensitivity/specificity for CT and MRI of 36/76%, and 71/91% for MRI, respectively).

Because of reclassification, CEUS led to a more appropriate categorization of CRC, as shown by two main results. First, CEUS met the expected per-category CDR better than CT/MRI,^17^ *i.e.* 11.1 *vs* 27.8% for category 2F, 77.8 *vs* 40% for category 3, 80 *vs* 75% for category 4, and 75 *vs* 0% for category 5, respectively. Second, CEUS was able to reclassify all IL findings, analogously to what reported by Bertolotto et al,^3^ suggesting that there is quite low likelihood for an incidental CT/MRI finding to remain indeterminate after CEUS. Not surprisingly, the agreement between CT/MRI and CEUS was slight (*k* = 0.12) and fair (*k* = 0.33) when assessing all lesions and CRC, respectively. One might assume that better agreement between CEUS and CT/MRI found in prior studies^9,25–27^ might has been overinflated by the inclusion of Bosniak 1 and 2 CT/MRI findings.

Was the use of CEUS effective from a clinical point of view? CEUS led to one false-negative case initially categorized 2F, which was assessed as a slow-growing cancer during the follow-up. On the other hand, the examination correctly downgraded 16.7% cases to category ≤2F. Our results suggest that CEUS has the potential to discontinue improper follow-up examinations, though long-term controls might be advisable to identify false-negative 2F assignments, which amount to up to 12% in literature^17^ (11% in our series). Further studies should define the most adequate time interval for follow-up, and/or assess which imaging and/or clinical features can stratify 2F patients for differentiating follow-up strategies. As testified by high specificity, a few cases only were false-positives, including one oncocytoma and one focal segmental glomerulosclerosis. However, CEUS enhancing pattern of oncocytoma, angiomyolipoma and other benign entities cannot reliably differentiate them from RCC,^28^ so that final diagnosis after surgical resection cannot be reasonably interpreted as an overtreatment. Other two suspicious lesions under surveillance showed no evolution during the follow-up. While classified as false-positives for the purpose of analysis, they might in fact represent low-grade cancers with slow evolution candidate to CEUS surveillance. Future studies on advanced imaging techniques, *e.g.* those based on artificial intelligence, might provide additional criteria for diagnosis in those doubtful cases in which biopsy or intervention cannot be performed.

There are some study limitations. Previous CT/MRI examinations were performed with different machines and techniques, even outside of our Institution, including protocols not targeted to the kidneys and the urinary tract. Additionally, different radiologists read the examinations. One might argue that those factors might have limited the robustness of initial assessment of IL and CRC, and in turn deflated the accuracy of CT and MRI. Of note, this reflects the incidental nature of the findings under investigation, and the real clinical scenario in which CEUS has been used as a problem-solving tool, *i.e.* a referral centre with a dedicated radiologist. This might represent a reasonable base for generalizability, at least in a similar ideal scenario, as indirectly proven by diagnostic accuracy we observed. However, we acknowledge that it was impossible to assess the inter-reader agreement, so that further multireaders studies on larger populations should validate our results. Second, having involved a single reader who performed both original CEUS examinations and retrospective rereading might have increased the risk of recall bias. However, we believe this risk was reasonably minimized by the long interval of time between original CEUS examinations (February 2016–October 2019) and re-evaluation, which started in late 2020. Third, we cannot exclude selection bias towards more aggressive lesions, as testified by 83.3% cancer prevalence in findings initially assessed as IL by CT/MRI, which is higher than previously observed (62%).^3^ The CDR we observed in Bosniak 3 findings was also higher than currently assumed to be within this category.^6^ Recent data suggest that many category three lesions are benign or slow-progressing entities.^17,29,30^ However, even assuming having preselected lesions at higher risk, this would be an ideal scenario in clinical practice, as the use of an additional imaging tool such as CEUS should be supported by adequate pre-test probability to be reasonably cost-effective. Finally, there is no histological diagnosis of benignity for most findings categorized ≤2F by CEUS, suggesting potential overestimation of CEUS sensitivity. On the other hand, the follow-up was reasonably long in our series, exceeding the 20 months average time to progression found by Tames AVC et al.^31^

In conclusion, CEUS was accurate in characterizing incidental CT/MRI renal findings initially classified as IL or CRC with Bosniak category 2F or larger. In particular, CEUS reclassified 100% IL, and 33.3% CRC compared to CT/MRI, referring most of them to proper treatment. Because of higher sensitivity for malignancy, our results suggest that CEUS is a valuable problem-solving tool to action more proper strategies of incidental CT/MRI observations, including follow-up discontinuation or intervention, with a minimal risk of false-negatives. Whether CEUS can be used systematically should be the matter for further studies stratifying patients’ pre-test risk of malignancy.
